# Synthesis and Evaluation of New Potential Benzo[*a*]phenoxazinium Photosensitizers for Anticancer Photodynamic Therapy

**DOI:** 10.3390/molecules23061436

**Published:** 2018-06-13

**Authors:** Juan Zhang, Wellington Tavares de Sousa Júnior, Victor Carlos Mello da Silva, Mosar Correa Rodrigues, José Athayde Vasconcelos Morais, Jia-Li Song, Zhi-Qiang Cheng, João Paulo Figueiró Longo, Ricardo Bentes Azevedo, Cheng-Shi Jiang, Luís Alexandre Muehlmann, Hua Zhang

**Affiliations:** 1 School of Biological Science and Technology, University of Jinan, Jinan 250022, China; zjandzq@163.com (J.Z.); 13285419800@163.com (J.-L.S.); czq13515312897@163.com (Z.-Q.C.); 2 Faculty of Ceilandia, University of Brasília, Brasilia 72220275, Brazil; wellingtonjunior123@hotmail.com (W.T.d.S.J.), victooormellooo@gmail.com (V.C.M.d.S.); mosarcr@gmail.com (M.C.R.); joseathayde_9@hotmail.com (J.A.V.M.);; 3 Institute of Biological Sciences, University of Brasília, Brasilia 70910900, Brazil; jplongo82@gmail.com (J.P.F.L.), razevedo@unb.br (R.B.A.)

**Keywords:** benzo[*a*]phenoxazinium, photosensitizer, reactive oxygen species, photodynamic therapy, anticancer

## Abstract

The use of photodynamic therapy (PDT) and development of novel photosensitizers (PSs) for cancer treatment have received more and more attention nowadays. In the present work, five benzo[*a*]phenoxazinium derivatives have been prepared and evaluated for their in vitro anticancer photodynamic activity for the first time. They are red light absorbers and show low fluorescence quantum yield. Of these compounds, **PS4** exhibited a higher quantum yield for reactive oxygen species (ROS) generation. The assays with cells in vitro showed that **PS1** and **PS4** were not significantly toxic in the dark, but was robustly toxic against the murine breast adenocarcinoma cells 4T1 and normal murine fibroblast cells NIH-3T3 upon photoactivation. More interestingly, **PS5** was particularly selective towards 4T1 cancer cells and nearly non-phototoxic to non-cancerous NIH-3T3 cells. The results described in this report suggest that these new benzo[*a*]phenoxazinium derivatives are potential candidates as PSs for anticancer PDT. Further investigation of benzo[*a*]phenoxaziniums for anticancer PDT is warranted.

## 1. Introduction

Photodynamic therapy (PDT) is a minimally invasive protocol that has been used in anticancer therapy for a long time [1]. PDT is based on the focal photoactivation of photosensitizers (PSs), which can directly act on the target tissues and then elicit photochemical reactions that eventually lead to oxidative stress [2]. The main consequences of these events include direct cytotoxicity, collapse of the tumor microvasculature, and/or activation of immune response against tumor antigens [3]. A particularly important benefit of PDT as a cancer therapy is the possibility to restrict its effects to the irradiated site, sparing the normal tissues. Although PDT has been successfully applied in the treatment of skin, gynecological, gastrointestinal, and some head and neck cancers for a long time, only a few PSs (e.g., porfimer sodium, temoporfin, aminolevulinic acid and photofrin) have been put into the market [4,5]. Up to now, most current work focuses on the improvement of porphyrin and chlorin-type PS molecules and, especially, synthesis of new type of PSs with high water solubility, strong absorption in near-infrared region, and long half-life [2,6].

**Figure 1:**
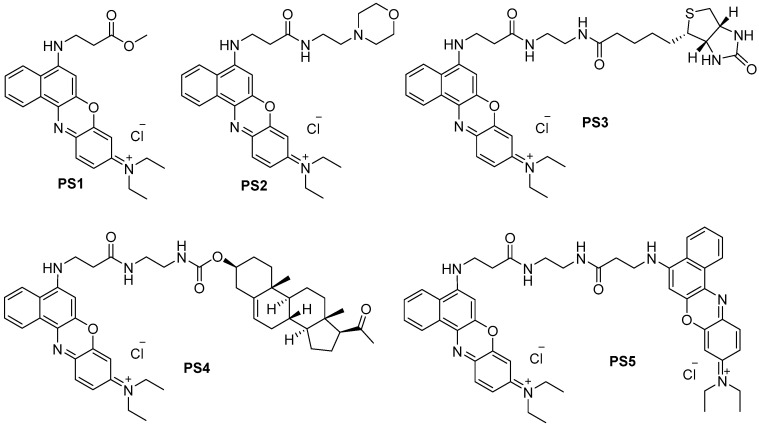
Structures of benzo[*a*]phenoxazinium chlorides **PS1**–**PS5**.

During our continuing project for developing new generation of PSs [7,8,9,10], the benzo[*a*]phenoxazinium chlorides dyes came to our attention due to their good photostability, high molar absorption, long-wavelength absorption, and relative low fluorescence quantum yield [11,12,13]. Literature survey indicated that benzo[*a*]phenoxazinium derivatives exhibited antifungal [12,14], and antimalarial activity [15], and especially functioned as PSs in antimicrobial PDT [15,16,17,18]. However, to our knowledge, the potential anticancer PDT of benzo[*a*]phenoxazinium dyes has been little investigated [19]. In this context, the main objective of the present work was to design and synthesize several new benzo[*a*]phenoxazinium chlorides **PS1**–**PS5** (Figure 1) and investigate their potential anticancer photodynamic activity.

## 2. Results and Discussion

### 2.1. Design and Synthesis

The ligand-mediated targeting (LMT) strategy in PDT has been explored to increase the efficacy and reduce adverse effects of PSs [2]. In the present study, five benzo[*a*]phenoxazinium chlorides possessing different functional fragments at the 5-amino position were designed and prepared based on the LMT strategy. **PS1** was a simple known benzo[*a*]phenoxazinium derivative with methyl propionate reported by Frade et al. [13,14,20], which was shown to have antimicrobial activity against *Saccharomyces cerevisiae* [14], but its anticancer PDT had not yet been investigated. Other compounds are new synthetic benzo[*a*]phenoxazinium derivatives: **PS2** possesses a morpholinoethylamine moiety, which is a well-known ligand for targeting lysosome [21]; **PS3** is equipped with a biotin moiety, which is a well-known tumor-targeting molecule [22] and has been used for selective delivery of PS to cancer tissues [23,24]; **PS4** was a conjugate of benzo[*a*]phenoxazinium and pregnenolone. Pregnenolone, known as a precursor to most hormones, had been explored as carrier of anticancer drugs [25]. In addition, several types of pregnenolone derivatives were reported to have anticancer activity by our and other groups [26,27,28]. **PS5** was synthesized as the first benzo[*a*]phenoxazinium dimer to determine the superimposed effect of the benzo[*a*]phenoxazinium core on anticancer PDT efficacy.

**Scheme 1:**
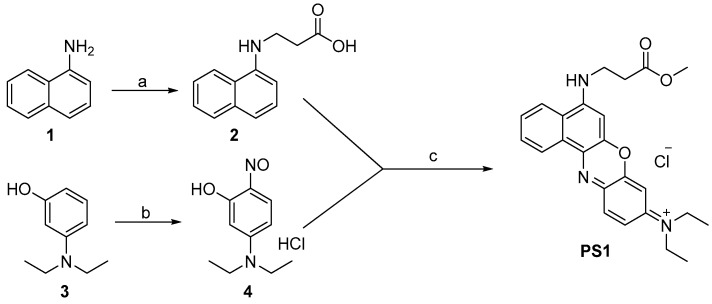
The synthesis of **PS1**. *Reagents and conditions*: (**a**) 3-bromopropanoic acid, Et_3_N, MeOH, reflux, overnight; (**b**) Cocn. HCl, sodium nitrite, H_2_O, 0–5 °C, 3.5 h; (**c**) Cocn. HCl, MeOH, reflux, 4 h.

**Scheme 2:**
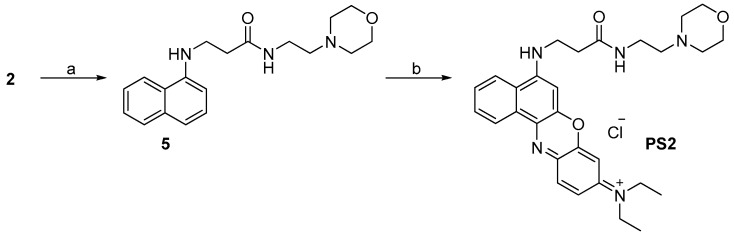
The synthesis of **PS2**. *Reagents and conditions*: (**a**) 2-morpholinoethanamine, HATU, iPrNEt_2_, CH_2_Cl_2_; (**b**) **4**, cocn. HCl, EtOH, reflux, 4 h.

**Scheme 3:**
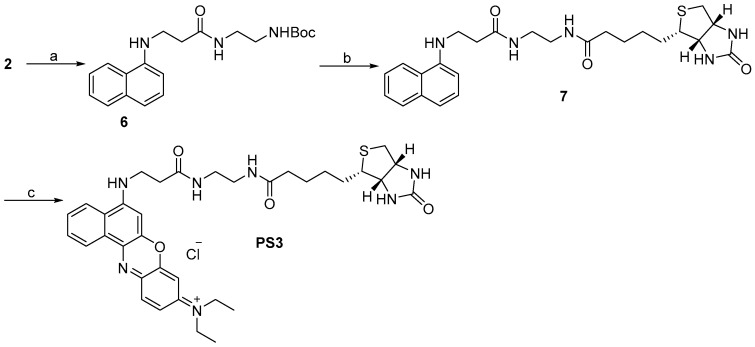
The synthesis of **PS3**. *Reagents and conditions*: (**a**) *tert*-butyl (2-aminoethyl)carbamate, HATU, iPrNEt_2_, CH_2_Cl_2_; (**b**) i. trifluoroacetic acid, CH_2_Cl_2_, 0 °C, overnight; ii. Biotin-NHS, Et_3_N, CH_3_CN, r.t., overnight; (**c**) **4**, EtOH, 5 drops of cocn. HCl, reflux, 4 h.

**Scheme 4:**
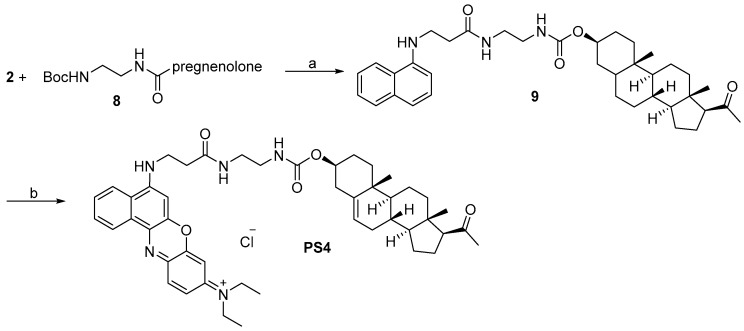
The synthesis of **PS4**. *Reagents and conditions*: (**a**) i. trifluoroacetic acid, CH_2_Cl_2_, 0 °C, overnight; ii. **2**, HATU, iPrNEt_2_, CH_2_Cl_2_, overnight.

**Scheme 5:**
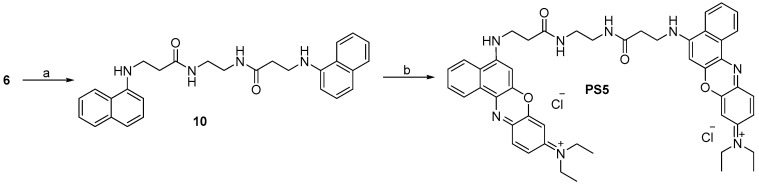
The synthesis of **PS5**. *Reagents and conditions*: (**a**) i. trifluoroacetic acid, CH_2_Cl_2_, 0 °C, overnight; ii **2**, HATU, iPrNEt_2_, CH_2_Cl_2_, overnight; (**b**) **4**, EtOH, 5 drops of cocn. HCl, reflux, 4 h.

The synthetic routes for target compounds **PS1** to **PS5** are depicted in Scheme 1, Scheme 2, Scheme 3, Scheme 4 and Scheme 5, respectively. Briefly, compound **2** was synthesized by alkylation of 1-naphthylamine (**1**) with 3-bromopropanoic acid, and nitroso derivative **4** was prepared from the nitration reaction of 3-(diethylamino)phenol (**3**). Then a cyclization reaction of **2** and **4** (in refluxing methanol) produced target compound **PS1**. The coupling reaction of **2** with 2-morpholinoethanamine, *tert*-butyl (2-aminoethyl)carbamate, and pregnenolone derivative **8** yielded intermediates **5**, **6** and **9**, respectively. Deprotection of N-Boc in **6** followed by coupling with Biotin-NHS gave compound **7** or followed by reaction with **2** produced **10**. Starting from intermediates **5**, **7**, **9**, **10** and using compound **4** once again, the target benzo[a]phenoxaziniums **PS2** to **PS5** were finally obtained in the last step using a similar synthetic protocol as that of **PS1**. The ^1^H-, ^13^C-NMR, LR-MS and HR-MS spectra for **PS1**–**PS5** can be found in Supplementary materials.

### 2.2. Absorption and Emission Studies

**Figure 2:**
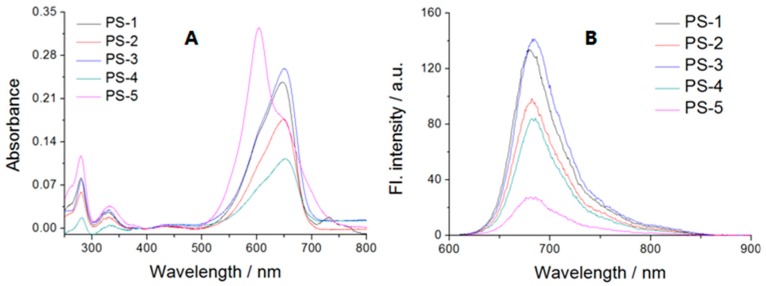
Absorption (**A**) and fluorescence ((**B**), excitation at λ 600 nm) spectra of **PS1**–**PS5** at 5 µM in water.

UV-vis absorption and emission spectra of 5 μM **PS1** to **PS5** in water were measured to investigate their optical properties (Table 1 and Figure 2A). The absorption maxima (λ_max_) for compounds **PS1** to **PS4** located at about 650 nm with the molar extinction coefficients (ε) between 22,600 and 51,800 M^−1^ cm^−1^, which can be ascribed to the π–π* transition of the large π system of the benzo[*a*]phenoxazinium core. **PS5** showed a shoulder peak at 649 nm (ε = 35,600 M^−1^ cm^−1^) from the benzo[*a*]phenoxazinium fluorophore. Meanwhile, **PS5** also showed a main absorption at 604 nm with a higher ε of 65,000 M^−1^ cm^−1^, which probably resulted from the π–π interaction of the two intramolecular benzo[*a*]phenoxazinium fluorophores as a result of the flexibility of the amide linker [29].

Under excitation at 600 nm, all these compounds exhibited near-infrared emissions at about 681 nm, with Stokes shifts of about 35 nm (Figure 2B). In addition, the relative fluorescence quantum yields (φ) were measured in water using fluorescein as a standard (φ_s_ = 0.98) [30]. These compounds all showed relatively low fluorescence quantum yields (0.025 to 0.116), which is consistent with the result of previously reported benzo[*a*]phenoxazinium chlorides [13]. This result indicates that **PS1**–**PS5** may not tend to decay back to the ground state by emitting fluorescence after excitation and have a great potential in undergoing intersystem crossing to form a relatively long-lived triplet state, which is necessary for acting as desirable PDT candidates.

### 2.3. ROS Production

**Figure 3:**
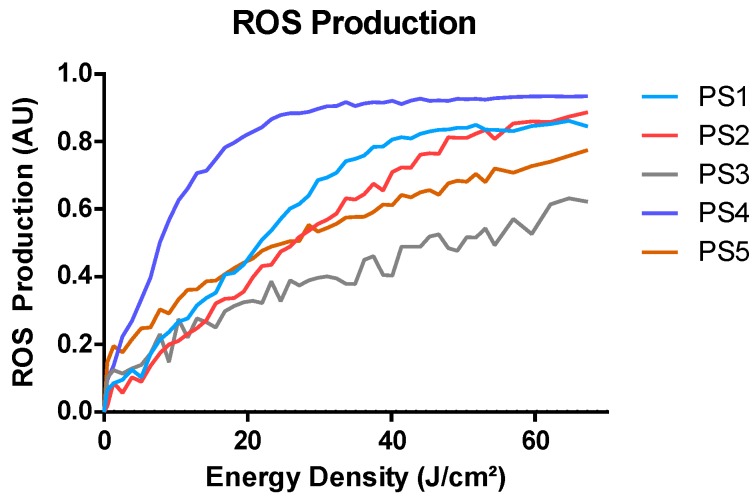
Photoactivated production of ROS by the candidate photosensitizers (**PS1** to **PS5**, 40 µM in water) expressed as arbitrary units (AU).

The reactive oxygen species (ROS) generated in photoreactions are the key factor for PDT as they can induce cytotoxicity via damage to different biomolecules, including proteins, nucleic acids and lipids [31,32]. Therefore, compounds **PS1** to **PS5** were evaluated for their effects on the production of ROS by DPBF method [7]. Figure 3 shows the results of ROS production when the compounds were irradiated with different energy densities. All these molecules produced ROS in an energy-dependent fashion, indicating that they are potential candidates for anticancer PDT. Remarkably, the photoactivated production of ROS was most intense with **PS4**, suggesting that this compound has the highest quantum yield for ROS generation. **PS3**, on the contrary, showed the lowest production of ROS compared with others.

### 2.4. Photodynamic Activity Against Cells In Vitro

**Figure 4:**
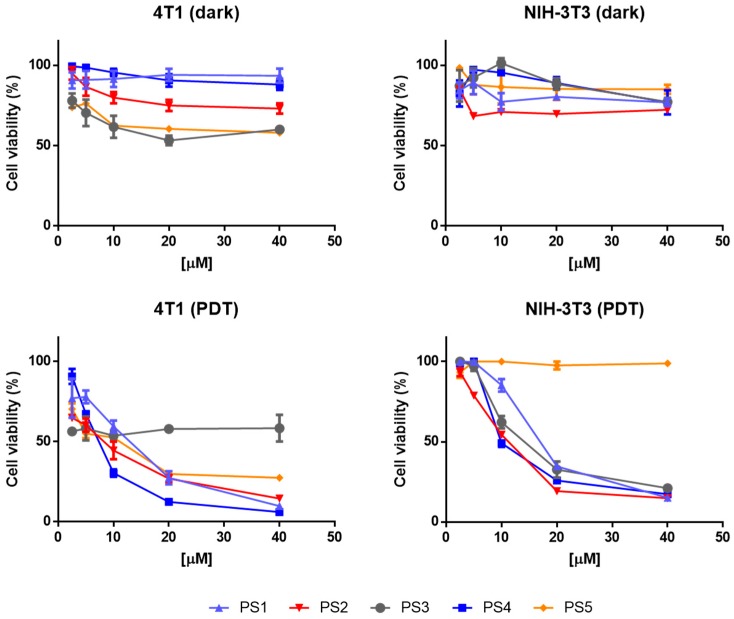
Toxicity of the compounds **PS1** to **PS5** at different concentrations against 4T1 and NIH-3T3 cells treated in the dark or irradiated with red light (λ 660 nm, 25.8 J/cm^2^).

Finally, in vitro photodynamic activities of **PS1** to **PS5** against two cell lines, including murine breast adenocarcinoma cell 4T1 and normal murine fibroblast cell NIH-3T3, were tested by irradiated (PDT) with 25.8 J/cm^2^ of light (λ 660 nm) or not (dark) by MTT bioassay [33]. As shown in Figure 4, all the tested compounds did not display significant toxicity towards both NIH-3T3 and 4T1 cells in the dark with inhibition ratio less than 50% within the concentration range of 2.5 to 40 μM. The absence of toxicity in the dark is a requirement for a desirable PS, as it avoids that non-irradiated tissues become affected during a PDT protocol. Regarding the photodynamic activity, it is remarkable that both **PS1** and **PS4** were robustly phototoxic against 4T1 cancer cell in a concentration-dependent manner (*p* < 0.05). Compound **PS3** presented no evident photodynamic activity against 4T1 cells. Interesting results were also obtained with **PS5**, which showed a significant phototoxicity against 4T1 cells but no activity against NIH-3T3 in PDT experiment, indicating that this compound might have a better selectivity towards the cancerous 4T1 cells tested in this study. However, further investigation on the selectivity of **PS5** towards cancerous cells and the mechanism of action is warranted.

## 3. Materials and Methods

### 3.1. General Information

Dimethyl sulfoxide (DMSO), 1,3-diphenylisobenzofuran (DPBF) and Kholiphor^®^ HS were obtained from Sigma (St. Louis, Missouri, USA). Roswell Park Memorial Institute (RPMI) medium and Dulbecco’s modified Eagle medium (DMEM) were obtained from Gibco (Waltham, MA, USA). The 3-(4,5-dimethylthiazol-2yl)-2,5-diphenyltetrazolium bromide (MTT) was purchased from Invitrogen (Carlsbad, CA, USA). The phosphate buffered saline (PBS) was supplied by Laborclin (Rio de Janeiro, Brazil). Commercially available reagents were used without further purification. Organic solvents were evaporated with reduced pressure using Büchi evaporators. Reactions were monitored by TLC using Yantai JingYou (Yantai, China) GF254 silica gel plates. Silica gel column chromatography was performed on an Isolera One system (Biotage, Uppsala, Sweden), and silica gel (200–300 mesh) from Qingdao Hailang Inc. (Qingdao, China). NMR spectra were measured on an Avance III 600 MHz spectrometer (Bruker, Fällanden, Switzerland). Chemical shifts were expressed in *δ* (ppm) and coupling constants (*J*) in Hz using solvent signals as internal standards (CDCl_3_, *δ*_H_ 7.26 ppm and *δ*_C_ 77.2 ppm; CD_3_OD, *δ*_H_ 3.31 ppm and *δ*_C_ 49.0 ppm; d_6_-acetone, *δ*_H_ 2.05 ppm and *δ*_C_ 29.8 ppm). ESI-MS were recorded on a 1260–6460 Triple Quad LC/MS (Agilent, Waldbronn, Germany) and HR-ESI-MS data were acquired on an Agilent Q-TOF 6520 system.

### 3.2. Chemistry

#### 3.2.1. Synthesis of 3-(Naphthalen-1-ylamino)propanoic Acid (**2**, C_13_H_13_NO_2_)

A mixture of naphthalen-1-amine **1** (2.0 g, 14.0 mmol, 1 equiv), 3-bromopropanoic acid (2.1 g, 14.4 mmol, 1 equiv) and Et_3_N (1.6 g, 14.4 mmol, 1 equiv) in 10 mL EtOH was refluxed overnight. The solvent was then removed under reduced pressure. The crude product was purified by silica gel flash chromatography using CH_2_Cl_2_/MeOH (10:1) as eluent to give compound **2** as a white solid (1.0 g, 3.64 mmol, 26% yield). ^1^H-NMR (CD_3_OD): *δ* 7.95 (d, *J* = 7.9 Hz, 1H), 7.73 (d, *J* = 7.8 Hz, 1H), 7.41–7.37 (m, 2H), 7.29 (dd, *J* = 7.6, 8.1 Hz, 1H), 7.17 (d, *J* = 8.1 Hz, 1H), 6.63 (d, *J* = 7.6 Hz, 1H), 3.56 (t, *J* = 6.8 Hz, 2H), 2.74 (t, *J* = 6.8 Hz, 2H). ^13^C-NMR (CD_3_OD): *δ* 176.3, 144.8, 135.9, 129.3, 127.6, 126.6, 125.4, 125.3, 121.7, 118.2, 105.2, 40.8, 34.4. ESI-MS m/z [M + H]^+^ calcd for C_13_H_14_NO_2_^+^ 216.1, found 216.2.

#### 3.2.2. Synthesis of 5-(Diethylamino)-2-nitrosophenol Hydrochloride (**4**, C_10_H_15_ClN_2_O_2_)

A solution of 3-diethylaminophenol **3** (3.3 g, 20 mmol, 1 equiv) in a mixture of concentrated HCl (7 mL) and water (7 mL) was cooled to 0 °C, and then a solution of sodium nitrite (1.4 g, 20 mmol, 1 equiv) in water (10 mL) was added dropwise to the above mixture. The reaction was stirred at 0–5 °C for 3.5 h to give a brown slurry. The slurry was filtered and washed with 4M aqueous HCl (6 mL) to give compound **4** as a brown solid (3.0 g, 13 mmol, 65% yield). This compound was used in the next step without further purification. ^1^H-NMR (CD_3_Cl): *δ* 7.37 (d, *J* = 8.0 Hz, 1H), 6.55 (m, 1H), 5.70 (d, *J* = 4.2 Hz, 1H), 3.50 (q, *J* = 8.0 Hz, 4H), 1.29 (t, *J* = 8.0 Hz, 6H). ^13^C- NMR (CD_3_Cl): *δ* 169.6, 157.4, 149.8, 135.8, 113.6, 96.3, 46.2, 13.5. ESI-MS m/z [M + H]^+^ calcd for C_10_H_15_N_2_O_2_^+^ 195.1, found 195.2.

#### 3.2.3. Synthesis of *N*-Ethyl-*N*-(5-(3-methoxy-3-oxopropylamino)-9*H*-benzo[*a*]phenoxazin-9-ylidene)ethanaminium Chloride (**PS1**, C_24_H_26_ClN_3_O_3_)

To a cold (ice bath) solution of **4** (160 mg, 0.6 mmol, 1.2 equiv) in MeOH (10 mL) was added **2** (100 mg, 0.5 mmol, 1 equiv) and 5 drop of concentrated HCl. The mixture was refluxed for 4 h. The solution was evaporated and purified by flash chromatography using CH_2_Cl_2_/MeOH (10:1) as eluent to give **PS1** as a blue solid (45 mg, 0.12 mmol, 24% yield). ^1^H-NMR (CD_3_OD): *δ* 8.83–8.80 (m, 1H), 8.29 (d, *J* = 8.0 Hz, 1H), 7.88 (dd, *J* = 7.2, 8.0 Hz, 1H), 7.81 (dd, *J* = 5.0, 9.3 Hz, 1H), 7.77 (dd, *J* = 7.2, 8.2 Hz, 1H), 7.28 (d, *J* = 9.3 Hz, 1H), 6.96 (s, 1H), 6.89 (s, 1H), 3.99 (t, *J* = 6.7 Hz, 2H), 3.75 (s, 3H), 3.71 (q, *J* = 7.2 Hz, 4H), 2.96 (t, *J* = 6.7 Hz, 2H), 1.35 (t, *J* = 7.2 Hz, 6H). ^13^C-NMR (CD_3_OD): *δ* 173.2, 159.1, 155.9, 153.1, 149.9, 134.7, 134.2, 132.9, 132.6, 132.1, 130.8, 125.2, 124.7, 123.8, 117.0, 97.0, 94.4, 52.5, 47.1, 41.4, 33.6, 13.0. ESI-MS *m*/*z* [M]^+^ 404.3. HR-ESIMS: [M]^+^ calcd for C_24_H_26_N_3_O_3_^+^ 404.1969, found 404.1967.

#### 3.2.4. Synthesis of *N*-(2-Morpholinoethyl)-3-(naphthalen-1-ylamino)propanamide (**5**, C_19_H_25_N_3_O_2_)

Compound **2** (100 mg, 0.5 mmol, 1 equiv) was dissolved in CH_2_Cl_2_ (10 mL), and then 2-morpholinoethanamine (120 mg, 0.9 mmol, 1.8 equiv), HATU (200 mg, 0.5 mmol, 1 equiv) and *i*PrNEt_2_ (0.2 mL, 1 mmol, 2 equiv) was added. The mixture was stirred overnight, and then concentrated. The residue was purified by silica gel chromatography with petroleum ether/acetone (1:1) as eluent to give compound **5** as a white solid (128 mg, 0.42 mmol, 84% yield). ^1^H-NMR (CDCl_3_): *δ* 7.83 (d, *J* = 8.0 Hz, 1H), 7.78 (d, *J* = 9.2 Hz, 1H), 7.45–7.41 (m, 2H), 7.35 (dd, *J* = 7.5, 8.2 Hz, 1H), 7.25 (d, *J* = 8.2 Hz, 1H), 6.54 (d, *J* = 7.5 Hz, 1H), 6.27 (brs, 1H), 5.07 (brs, 1H), 3.64 (t, *J* = 6.0 Hz, 2H), 3.50 (brs, 4H), 3.32 (t, *J* = 6.0 Hz, 2H), 2.64 (d, *J* = 6.0 Hz, 2H), 2.38 (t, *J* = 6.0 Hz, 2H), 2.29 (brs, 4H)*.* ^13^C- NMR (CDCl_3_): *δ* 171.8, 142.9, 134.4, 128.6, 126.5, 125.8, 124.8, 123.7, 120.0, 117.7, 104.4, 66.7, 56.8, 53.2, 40.3, 35.5, 35.3*.* ESI-MS *m*/*z*: [M + H]^+^ calcd for C_19_H_25_N_3_O_2_^+^ 328.2, found 328.3.

#### 3.2.5. Synthesis of *N*-Ethyl-*N*-(5-(3-(2-morpholinoethylamino)-3-oxopropylamino)-9*H*-benzo[*a*]phenoxazin-9-ylidene)ethanaminium Chloride (**PS2**, C_29_H_36_ClN_5_O_3_)

To a cold solution of compound **4** (50 mg, 0.2 mmol, 1 equiv) in EtOH (5 mL) was added compound **5** (60 mg, 0.2 mmol, 1 equiv) and 5 drops of concentrated HCl. The mixture was refluxed for 4 h. The solution was evaporated and purified by silica gel flash chromatography using CH_2_Cl_2_/MeOH (10:1) as eluent to give compound **PS2** as a blue solid (54 mg, 0.11 mmol, 55% yield). ^1^H-NMR (CD_3_OD): *δ* 8.72 (s, 1H), 8.39 (s, 1H), 7.84 (s, 1H), 7.75 (s, 2H), 7.25 (s, 1H), 6.85 (s, 1H), 4.04–3.96 (m, 6H), 3.70–3.66 (m, 8H), 3.34 (s, 2H), 3.18 (s, 2H), 2.91 (s, 2H), 1.35 (s, 6H). ^13^C-NMR (CD_3_OD): *δ* 174.1, 158.9, 155.8, 152.9, 149.7, 134.5, 134.2, 132.9, 132.3, 131.9, 130.8, 125.4, 124.5, 124.2, 117.0, 96.9, 94.6, 64.8, 58.1, 53.4, 41.8, 35.1, 34.7, 13.0. ESI-MS *m*/*z* [M]^+^ 502.2. HR-ESIMS: [M]^+^ calcd for C_29_H_36_N_5_O_3_^+^ 502.2813, found 502.2812; [M + H]^2+^ calcd for C_29_H_36_N_5_O_3_^2+^ 251.6442, found 251.6445.

#### 3.2.6. Synthesis of *tert*-Butyl 2-(3-(naphthalen-1-ylamino)propanamido)ethylcarbamate (**6**, C_20_H_27_N_3_O_3_)

Compound **2** (100 mg, 0.5 mmol, 1 equiv) was dissolved in CH_2_Cl_2_ (10 mL), and then *tert*-butyl (2-aminoethyl)carbamate (149 mg, 1.0 mmol, 2 equiv), HATU (200 mg, 0.5 mmol, 1 equiv) and *i*PrNEt_2_ (0.2 mL, 1 mmol, 2 equiv) was added. The mixture was stirred overnight, and then concentrated. The residue was purified by silica gel chromatography with CH_2_Cl_2_/MeOH (10:1) as eluent to give compound **6** as a white solid (160 mg, 0.48 mmol, 96% yield). ^1^H-NMR (CDCl_3_): *δ* 7.91–7.89 (m, 1H), 7.79–7.77 (m, 1H), 7.45–7.41 (m, 2H), 7.46–7.43 (m, 1H), 7.34 (dd, *J* = 7.6, 8.0 Hz, 1H), 7.27 (d, *J* = 7.0 Hz, 1H), 6.6 (d, *J* = 7.6 Hz, 1H), 6.48 (brs, 1H), 4.85 (brs, 1H), 3.61 (t, *J* = 6.0 Hz, 2H), 3.37–3.34 (m, 2H), 3.26–3.24 (m, 2H), 2.63 (t, *J* = 6.0 Hz, 2H), 1.41 (s, 9H)*.* ^13^C-NMR (CDCl_3_): *δ* 172.6, 157.2, 142.9, 134.5, 128.7, 126.6, 126.0, 125.1, 124.0, 120.3, 118.2, 105.0, 80.0, 41.1, 40.8, 40.3, 35.2, 28.4*.* ESI-MS *m*/*z*: [M + H]^+^ calcd for C_20_H_28_N_3_O_3_^+^ 484.2, found 358.0.

#### 3.2.7. Synthesis of *N*-(2-(3-(Naphthalen-1-ylamino)propanamido)ethyl)-5-(2-oxohexahydro-1*H*-thieno[3,4-*d*]imidazol-4-yl)pentanamide (**7**, C_25_H_33_N_5_O_3_S)

To a cold (ice bath) solution of **6** (120 mg, 0.34 mmol, 1 equiv) in CH_2_Cl_2_ (5 mL) was added trifluoroacetic acid (100 μL, 1 mmol, 2.9 equiv) under 0 °C. The mixture was stirred overnight, and then concentrated to give a residue, which was used in the next step without further purification. The obtained residue was dissolved in CH_3_CN (5 mL), and then Biotin-NHS (172 mg, 0.50 mmol, 1.5 equiv), and Et_3_N (0.2 mL, 1 mmol, 2.9 equiv) were added. The mixture was stirred overnight, and then concentrated. The residue was purified by silica gel chromatography with CH_2_Cl_2_/MeOH (10:1) as eluent to give compound **7** as a white solid (85 mg, 0.18 mmol, 52% yield). ^1^H-NMR (DMSO-*d*_6_): *δ* 8.08 (d, *J* = 8.3 Hz, 1H), 7.99 (brs, 1H), 7.81 (brs, 1H), 7.75 (d, *J* = 8.3 Hz, 1H), 7.43 (ddd, *J* = 1.0, 6.7, 8.4 Hz, 1H), 7.39 (ddd, *J* =1.4, 6.8, 9.0 Hz, 1H), 7.29 (dd, *J* = 7.8, 7.9 Hz, 1H), 7.11 (d, *J* = 8.2 Hz, 1H), 6.54 (d, *J* = 8.6 Hz, 1H), 6.42 (s, 1H), 6.35 (s, 1H), 6.19 (dd, *J* = 5.4, 5.5 Hz, 1H), 4.28–4.26 (m, 1H), 4.11–4.09 (m, 1H), 3.44–3.41 (m, 1H), 3.31 (m, 5H), 2.78 (dd, *J* = 5.1, 12.5 Hz, 1H), 2.56(d, *J* = 12.5 Hz, 1H), 2.04 (t, *J* = 1.1 Hz, 1H), 1.61–1.57 (m, 1H), 1.53–1.43 (m, 3H), 1.32–1.23 (m, 2H), 1.17 (t, *J* = 7.3 Hz, 1H)*.*^13^C-NMR (DMSO-*d*_6_): *δ* 172.4, 171.1, 162.7, 143.8, 134.0, 127.9, 126.8, 125.6, 124.0, 123.0, 121.4, 115.6, 102.9, 61.0, 59.2, 55.4, 45.7, 40.1, 38.5, 38.3, 35.3, 34.8, 28.2, 28.0, 25.2*.* ESI-MS *m*/*z*: [M + H]^+^ calcd for C_25_H_34_N_5_O_3_S^+^ 484.2, found 484.3.

#### 3.2.8. Synthesis of *N*-Ethyl-*N*-(5-(3-oxo-3-(2-(5-(2-oxohexahydro-1*H*-thieno[3,4-*d*]imidazol-4-yl)pentanamido)ethylamino)propylamino)-9*H*-benzo[*a*]phenoxazin-9-ylidene)ethanaminium Chloride (**PS3**, C_35_H_44_ClN_7_O_4_S)

To a cold solution of **4** (50 mg, 0.2 mmol, 1 equiv) in EtOH (5 mL) was added **7** (60 mg, 1.2 mmol, 6 equiv) and 5 drops of concentrated HCl. The mixture was refluxed for 4 h. The solution was evaporated and purified by silica gel flash chromatography using CH_2_Cl_2_/MeOH (10:1) as eluent to give **PS3** as a blue solid (53 mg, 0.12 mmol, 62% yield). ^1^H-NMR (CD_3_OD): *δ* 8.89–8.83 (m, 1H), 8.37 (d, *J* = 7.5 Hz, 1H), 7.89–7.79 (m, 2H), 7.75–7.55 (m, 2H), 7.29 (d, *J* = 9.0 Hz, 1H), 7.01 (s, 1H), 6.91 (s, 1H), 4.46 (s, 1H), 4.28 (s, 1H), 4.01 (s, 1H), 3.72–3.71 (m, 4H), 3.56 (s, 1H), 3.15(s, 1H), 2.89–2.74 (m, 2H), 2.66 (s, 1H), 2.15 (s, 1H), 1.67–1.51 (m, 4H), 1.35 (t, *J* = 7.0 Hz, 1H), 1.17–1.14 (m, 2H). ^13^C-NMR (CD_3_OD): *δ* 176.4, 173.2, 159.1, 155.8, 153.2, 149.8, 134.7, 134.3, 132.9, 132.6, 132.1, 130.9, 125.5, 124.7, 124.0, 121.7, 117.0, 97.0, 94.5, 63.3, 61.6, 56.9, 47.1, 42.0, 41.0, 40.4, 39.9, 36.7, 35.5, 29.7, 29.4, 26.7, 13.0. ESI-MS *m*/*z* [M]^+^ 658.2. HR-ESIMS: [M]^+^ calcd for C_35_H_44_N_7_O_4_S^+^ 658.3170, found 658.3171.

#### 3.2.9. Synthesis of (3*S*,10*R*,13*S*,17*S*)-17-Acetyl-10,13-dimethyl-2,3,4,7,8,9,10,11,12,13,14,15,16,17-tetradecahydro-1*H*-cyclopenta[a]phenanthren-3-yl 2-(3-(naphthalen-1-ylamino)propanamido)ethylcarbamate (**9**, C_37_H_49_N_3_O_4_)

To a solution of (3*S*,10*R*,13*S*,17*S*)-17-acetyl-10,13-dimethyl-2,3,4,7,8,9,10,11,12,13,14,15,16,17-tetradecahydro-1*H*-cyclopenta[*a*]phenanthren-3-yl 2-(*tert*-butoxycarbonylamino)ethylcarbamate (**8**, 300 mg, 0.6 mmol, 1 equiv) in CH_2_Cl_2_ (10 mL) was added trifluoroacetic acid (300 μL, 3 mmol, 5 equiv) under 0 °C. The mixture was stirred overnight, and then concentrated to give a residue, which was used in the next step without further purification. The above residue was dissolved in CH_2_Cl_2_, (10 mL) and then compound **2** (128 mg, 0.6 mmol, 1 equiv), HATU (248 mg, 0.7 mmol, 1.1 equiv) and *i*PrNEt_2_ (0.2 mL, 1 mmol, 2 equiv) were added. The mixture was stirred overnight, and then concentrated. The residue was purified by silica gel chromatography with petroleum ether/acetone (1:1) as eluent to give compound **9** as a white solid (205 mg, 0.34 mmol, 57%). ^1^H-NMR (CDCl_3_): *δ* 7.87 (d, *J* = 7.5 Hz, 1H), 7.78 (m, 2H), 7.46–7.42 (m, 2H), 7.34 (dd, *J* = 7.9, 7.8 Hz, 1H), 7.25 (d, *J* = 8.2 Hz, 1H), 6.62 (d, *J* = 7.5 Hz, 1H), 5.34 (s, 1H), 5.15 (s, 1H), 4.95 (s, 1H), 3.62–3.60 (m, 2H), 3.36 (d, *J* = 5.4 Hz, 1H), 2.62 (dd, *J* = 6.1, 6.1 Hz, 1H), 2.53 (dd, *J* = 9.1, 8.8 Hz, 1H), 2.20–2.14 (m, 3H), 2.12 (s, 3H), 2.05–1.96 (m, 2H), 1.83–1.82 (m, 2H), 1.68–1.43 (m, 9H), 1.25–1.21 (m, 1H), 1.15–1.09 (m, 1H), 0.98 (s, 3H), 0.62 (s, 3H). ^13^C-NMR (CDCl_3_): *δ* 209.8, 172.7, 157.3, 143.6, 139.8, 134.5, 128.7, 126.6, 126.0, 125.0, 123.9, 122.4, 120.3, 117.9, 104.6, 74.8, 63.8, 57.0, 50.0, 44.1, 40.9, 40.7, 40.5, 38.9, 38.6, 37.1, 36.7, 35.4, 31.9, 31.9, 31.7, 28.2, 24.6, 23.0, 21.1, 19.4, 13.4. ESI-MS *m*/*z*: [M + H]^+^ calcd for C_37_H_50_N_3_O_4_^+^ 600.4, found 600.1.

#### 3.2.10. Synthesis of *N*-(5-(3-(2-(((3*S*,10*R*,13*S*,17*S*)-17-Acetyl-10,13-dimethyl-2,3,4,7,8,9,10,11,12,13,14,15,16,17-tetradecahydro-1*H*-cyclopenta[*a*]phenanthren-3-yloxy)carbonylamino)ethylamino)-3-oxopropylamino)-9*H*-benzo[*a*]phenoxazin-9-ylidene)-*N*-ethylethanaminium Chloride (**PS4**, C_47_H_60_ClN_5_O_5_)

To a cold (ice bath) solution of compound **4** (30 mg, 0.13 mmol, 1.6 equiv) in EtOH (5 mL) was added compound **9** (50 mg, 0.08 mmol, 1 equiv) and 5 drops of concentrated HCl. The mixture was refluxed for 4 h. The solution was evaporated and purified by silica gel flash chromatography using CH_2_Cl_2_/MeOH (10:1) as eluent to give compound **PS4** as a blue solid (45 mg, 0.06 mmol, 69% yield). ^1^H-NMR (CD_3_OD): *δ* 8.82 (d, *J* = 11 Hz, 1H), 8.31 (dd, *J* = 8.3, 8.4 Hz, 1H), 7.88 (dd, *J* = 7.4, 7.6 Hz, 1H), 7.82 (d, *J* = 9.2 Hz, 1H), 7.79 (dd, *J* = 7.3, 7.6 Hz, 1H), 7.28 (d, *J* = 8.7 Hz, 1H), 6.99 (s, 1H), 6.89 (s, 1H), 5.02 (s, 1H), 4.23 (m, 1H), 3.99–3.97 (m, 2H), 3.72–3.69 (m, 4H), 2.79–2.77 (m, 2H), 2.55 (dd, *J* = 6.7, 7.0 Hz, 1H), 2.15–2.08 (m, 5H), 1.98 (d, *J* = 8.2 Hz, 1H), 1.73–1.60 (m, 6H), 1.83–1.82 (m, 2H), 1.38 (d, *J* = 6.7 Hz, 1H), 1.35 (t, *J* = 7.2 Hz, 1H), 0.82 (s, 3H), 0.52 (s, 3H). ^13^C-NMR (CD_3_OD): *δ* 212.1, 173.4, 159.0, 158.9, 155.7, 153.0, 149.7, 141.1, 141.1, 134.2, 132.9, 132.6, 130.9, 125.6, 125.5, 124.1, 124.0, 123.0, 116.8, 97.1, 94.7, 75.3, 64.6, 57.9, 51.2, 47.1, 44.9, 41.1, 39.7, 38.0, 37.5, 36.1, 35.6, 35.5, 33.0, 32.9, 32.6, 31.7, 29.1, 25.4, 23.7, 22.0, 19.6, 13.5, 13.0. ESI-MS *m*/*z*: [M]^+^ 774.4. HR-ESIMS: [M]^+^ calcd for C_47_H_60_N_5_O_5_^+^ 774.4589, found 774.4595.

#### 3.2.11. Synthesis of *N,N′*-(Ethane-1,2-diyl)bis(3-(naphthalen-1-ylamino)propanamide) (**10**, C_28_H_30_N_4_O_2_)

To a solution (ice bath) of compound **6** (170 mg, 0.5 mmol, equiv) in CH_2_Cl_2_ (5 mL) was added trifluoroacetic acid (500 μL, 5 mmol, 14.5 equiv) under 0 °C. The mixture was stirred overnight, and then concentrated to give a residue, which was used in the next step without further purification. The above residue was dissolved in CH_2_Cl_2_ (5 mL) and then compound **2** (102 mg, 0.5 mmol, 1 equiv), HATU (200 mg, 0.5 mmol, 1 equiv) and *i*PrNEt_2_ (0.2 mL, 1 mmol, 2 equiv) were added. The mixture was stirred overnight, and then concentrated. The residue was purified by silica gel chromatography with CH_2_Cl_2_/MeOH (20:1) as eluent to give compound **10** as a white solid (185 mg, 0.44 mmol, 87% yield). ^1^H-NMR (*d_6_*-acetone): *δ* 8.40 (d, *J* = 8.4 Hz, 1H), 7.76 (d, *J* = 8.4 Hz, 1H), 7.41–7.38 (m, 3H), 7.29 (dd, *J* = 7.9, 7.9 Hz, 1H), 7.15 (d, *J* = 8.2 Hz, 1H), 6.59 (d, *J* = 7.4 Hz, 1H), 5.84 (s, 1H), 3.52 (t, *J* = 6.4 Hz, 2H), 3.30 (t, *J* = 2.6 Hz, 2H), 2.57 (d, *J* = 6.4 Hz, 2H)*.* ^13^C-NMR (*d_6_*-acetone): *δ* 172.9, 144.9, 135.5, 129.1, 127.6, 126.4, 125.1, 124.6, 121.7, 117.2, 104.4, 41.1, 40.1, 35.7*.* ESI-MS *m*/*z*: [M + H]^+^ calcd for C_28_H_31_N_4_O_2_^+^ 455.2, found 455.1.

#### 3.2.12. Synthesis of di(*N*-(5-(3-(2-Aminoethylamino)-3-oxopropylamino)-9*H*-benzo[*a*]phenoxazin-9-ylidene))-*N,N′*-ethylethanaminium Dichloride (**PS5**, C_48_H_52_Cl_2_N_8_O_4_)

To a cold solution of compound **4** (125 mg, 0.5 mmol, 1 equiv) in EtOH (5 mL) was added compound **10** (100 mg, 0.2 mmol, 1 euqiv) and 5 drops of concentrated HCl. The mixture was refluxed for 4 h. The solution was evaporated and purified by silica gel flash chromatography using CH_2_Cl_2_/MeOH (10:1) as eluent to give compound **PS5** as a purple solid (80 mg, 0.22 mmol, 45% yield). ^1^H-NMR (CD_3_OD): *δ* 8.29 (d, *J* = 7.9 Hz, 1H), 8.26 (d, *J* = 8.0 Hz, 1H), 7.65 (dd, *J* = 7.3, 7.6 Hz, 1H), 7.57 (dd, *J* = 7.3, 9.3 Hz, 1H), 7.47 (d, *J* = 9.3 Hz, 1H), 7.11 (d, *J* = 9.2 Hz, 1H), 6.61 (s, 1H), 6.58 (s, 1H), 3.74 (s, 2H), 3.64 (q, *J* = 7.1 Hz, 4H), 3.40 (s, 2H), 2.81 (s, 2H), 1.34 (t, *J* = 7.1 Hz, 6H). ^13^C-NMR (CD_3_OD): *δ* 173.5, 158.4, 155.6, 152.2, 149.1, 134.0, 133.9, 132.8, 131.9, 131.5, 130.7, 125.1, 124.2, 124.2, 116.9, 96.9, 94.4, 47.2, 42.2, 40.3, 35.1, 13.1. ESI-MS *m*/*z* [M]^2+^ 402.3. HR-ESIMS: [M]^2+^ calcd for C_48_H_52_N_8_O_4_^2+^ 402.2050, found 402.2049.

### 3.3. General Spectroscopic Measurements

Absorption spectra were recorded in a Shimadzu UV-2600 Spectrophotometer (Shimadzu, Kyoto, Japan). Fluorescence measurements were performed using an Agilent Cary Eclipse (Varian, Palo Alto, California, USA). The concentrations of all the compounds were 5 µM. Fluorescence spectra were corrected for the instrumental response of the system. All solutions were prepared using Milli-Q grade water. The fluorescence quantum yields (φ) were determined according to the method Equation (1) below:

φ*_u_* = φ*_s_* × (F*_u_*/ F*_s_*) × (A*_s_* / A*_u_*) × (η*_u_*/ η*_s_*)^2^ (1)

where φ is fluorescence quantum yield; F is integrated area under the corrected emission spectra; η is the refractive index of the solution; A is the absorbance at the excitation wavelength; the subscripts u and s refer to the unknown and the standard, respectively. We chose fluorescein in water as reference, φ*_s_* = 0.98.

### 3.4. ROS Detection

To evaluate the capacity of the compounds to produce singlet oxygen, the DPBF method was used. Briefly, 200 µL-aliquots of compounds in DMSO (40 µM) were plated in 96-well plates. Then, 10 μL of a solution of DPBF in ethanol (0.22 mg/mL) were added to each aliquot. Controls consisted of each compound without DPBF, and DPBF alone. Then, the microplate was irradiated every 10 s using 660 nm LED (Light Emitting Diode, XL001WP01NRC660, Shenzhen S. O. Co, Shenzhen, China). The optical density of the DPBF solution at 414 nm was used as an index of ROS production, as the DPBF is degraded by ROS and its light absorption at this wavelength is thus decreased.

### 3.5. Phototoxicity Assay

The toxicity of different treatments against 4T1 and NIH-3T3 cells was measured by an MTT assay. Briefly, 4T1 and NIH-3T3, 1 × 10^4^ cells per well, were treated with different concentrations of the compounds for 30 min, in RPMI and DMEM, respectively, and then washed twice with PBS. After, the microplates were: (1) maintained in the dark; or (2) irradiated with a light emitting diode (LED, λ 660 nm) at a final energy density of 25.8 J/cm^2^. The control consisted of cells that received only culture medium. Next, the cells were washed with PBS, cultured for further 24 h, and then the culture medium was replaced by a 0.5 mg/mL MTT solution in culture medium. The cells were then incubated for 2.5 h at 37 °C in a 5% CO_2_, humid atmosphere. The MTT solution was then discarded, the formazan produced by the viable cells was extracted with 200 µL DMSO, and the optical density was read at λ 595 nm with a microplate spectrophotometer. This experiment was performed in triplicate for each treatment, and the results were expressed as percentages relative to control.

### 3.6. Statistical Analysis

Data were analyzed by one-way ANOVA, with Sidak’s post-test (α = 0.05). Analyzes were performed with GraphPad Prism^®^ 6.0 software (GraphPad Software, La Jolla, CA,).

## 4. Conclusions

In conclusion, four benzo[*a*]phenoxazinium derivatives **PS1**–**PS4** bearing different functional groups in the amino side chain and the first benzo[*a*]phenoxazinium dimer **PS5** were prepared. The investigation on optical properties of **PS1** to **PS5** in water indicated that they are red light absorbers with low fluorescence quantum yields (0.025~0.116). The ROS production study revealed that all these benzo[*a*]phenoxaziniums produced ROS in an energy-dependent fashion, with **PS4** having the highest ROS quantum yield. Finally, the anticancer PDT activities of this series of benzo[*a*]phenoxaziniums were evaluated for the first time. The bioassay results indicated that **PS1** and **PS4** show significant photodynamic activities against 4T1 cancer cells and NIH-3T3 normal murine fibroblast cells, and **PS5** showed intriguing anticancer PDT activity selectively towards 4T1 cancer cells over NIH-3T3 normal cells. Together with the optical properties and photodynamic bioassay results, this series of benzo[*a*]phenoxazinium derivatives can be highlighted as new PSs worthy of further investigation in anticancer PDT study.

## Figures and Tables

**Table 1:** Absorption and emission data of benzo[*a*]phenoxazinium chlorides **PS1**-**PS5** in water.

Compound	λ_abs_^a^	Ε ^b^	λ_em_^a^	Δλ ^a^	φ
**PS1**	648	47,400	681	33	0.075
**PS2**	649	35,400	682	33	0.077
**PS3**	650	51,800	684	34	0.082
**PS4**	651	22,600	686	35	0.116
**PS5**	649 (604)	35,600 (65,000)	686	37	0.025

^a^ nm; ^b^ M^−1^ cm^−1^.

## Supplementary Materials

The following are available online at http://www.mdpi.com/1420-3049/23/6/1436/s1, ^1^H-, ^13^C-NMR, LR-MS and HR-MS spectra for **PS1**–**PS5**.
