# Supplementary material for: Synthesis and Evaluation of New Potential Benzo[a]phenoxazinium Photosensitizers for Anticancer Photodynamic Therapy
Source: Molecules. 2018 Jun 13;23(6):1436. doi: 10.3390/molecules23061436 (PMC6100483; doi:10.3390/molecules23061436)

molecules

Supporting Information

Synthesis and evaluation of new potential benzo[*a*]phenoxazinium photosensitizers for anticancer photodynamic therapy

Juan Zhang[a,b], Wellington Tavares de Sousa Júnior[b], Victor Carlos Mello da Silva[b], Mosar Correa Rodrigues[b,c], José Athayde Vasconcelos Morais[b,c], Jia-Li Song[a], Zhi-Qiang Cheng[a], João Paulo Figueiró Longo[c], Ricardo Bentes Azevedo[c], Cheng-Shi Jiang[a,*], Luís Alexandre Muehlmann[b,*], Hua Zhang[a,*]

[a] *School of Biological Science and Technology, University of Jinan, Jinan 250022, China*
[b] *Faculty of Ceilandia, University of Brasília, Brasilia 72220275, Brazil*
[c] *Institute of Biological Sciences, University of Brasília, Brasilia 70910900, Brazil*

*Corresponding authors. E-mail addresses: jiangchengshi-20@163.com (C.-S. Jiang); luisalex@unb.br (L. A. Muehlmann); bio_zhangh@ujn.edu.cn (H. Zhang)

1. Spectra

Figure 1. 1H spectrum of **PS-1**

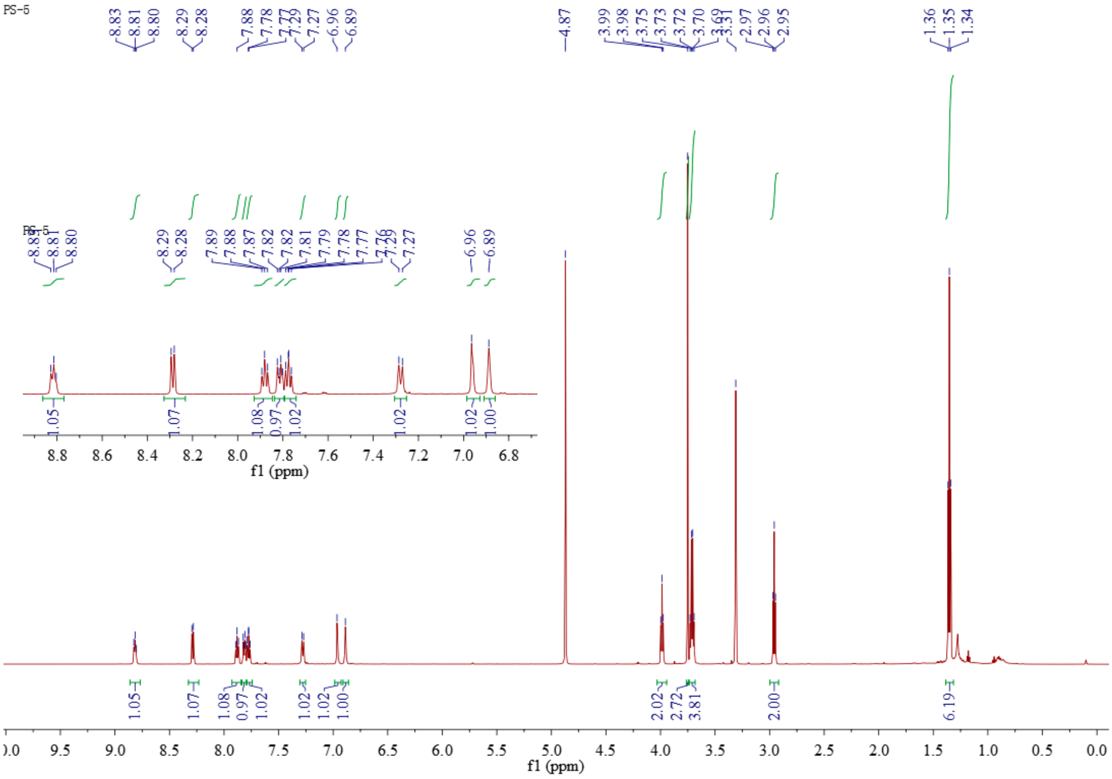

Figure 2. ^{13}C spectrum of **PS-1**

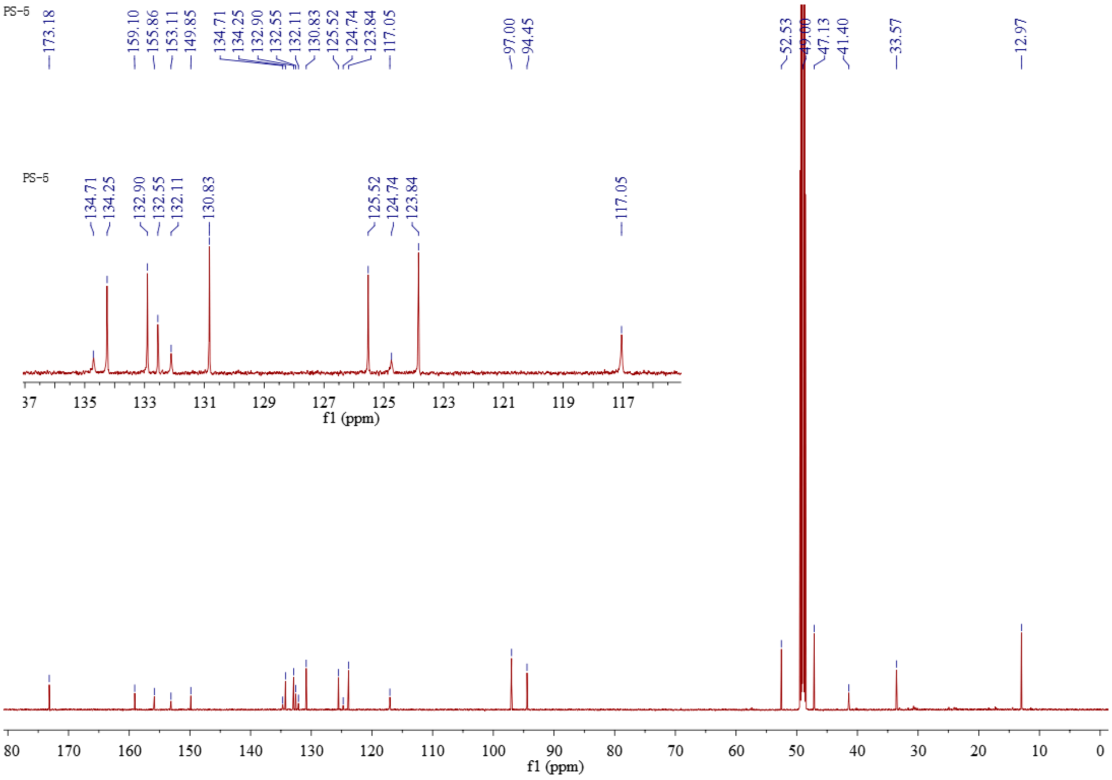

Figure 3. LR-MS spectrum of **PS-1**

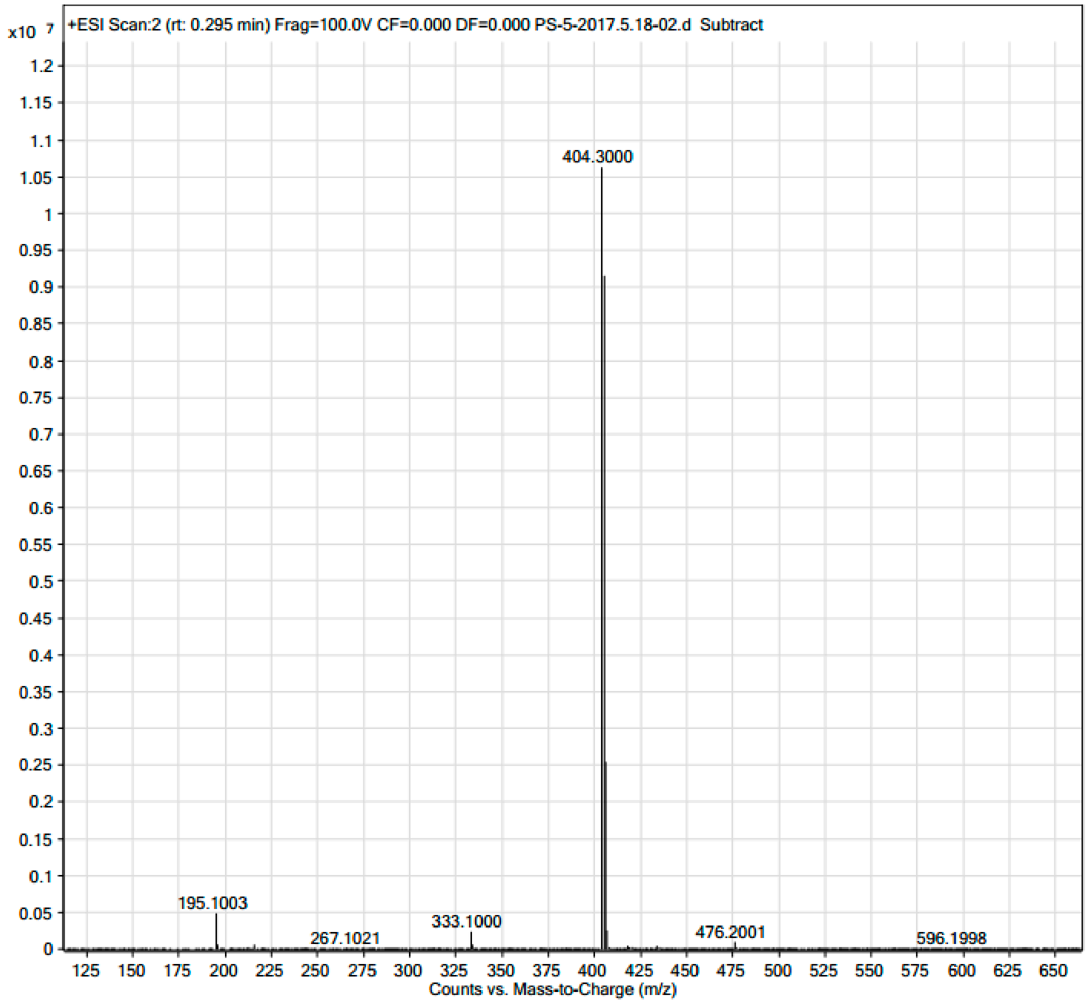

Figure 4. HR-MS spectrum of **PS-1**

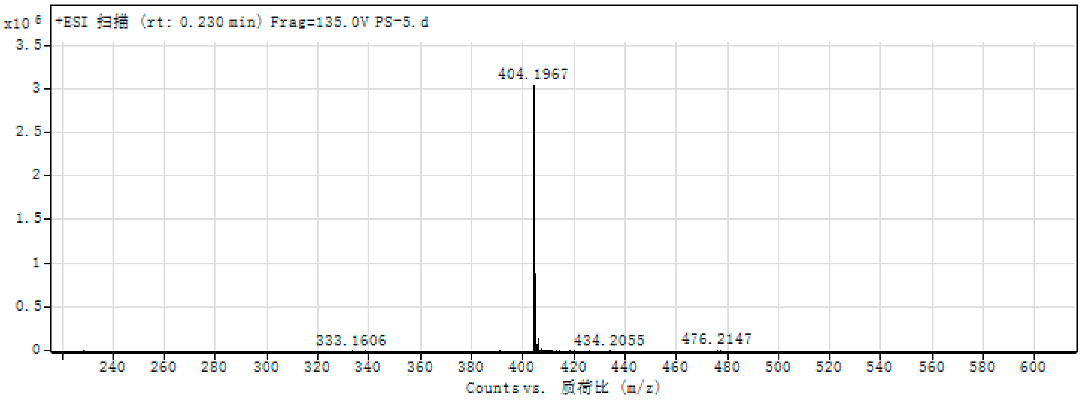

Figure 5. ^{1}H spectrum of **PS-2**

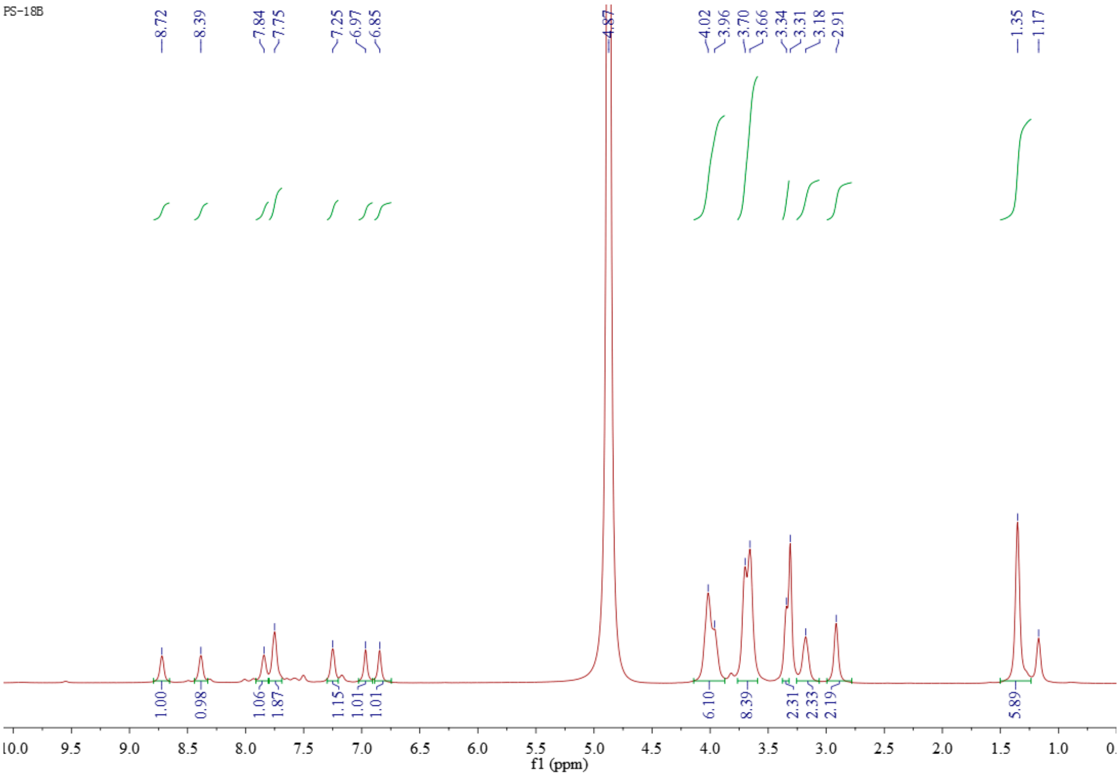

Figure 6. ^{13}C spectrum of **PS-2**

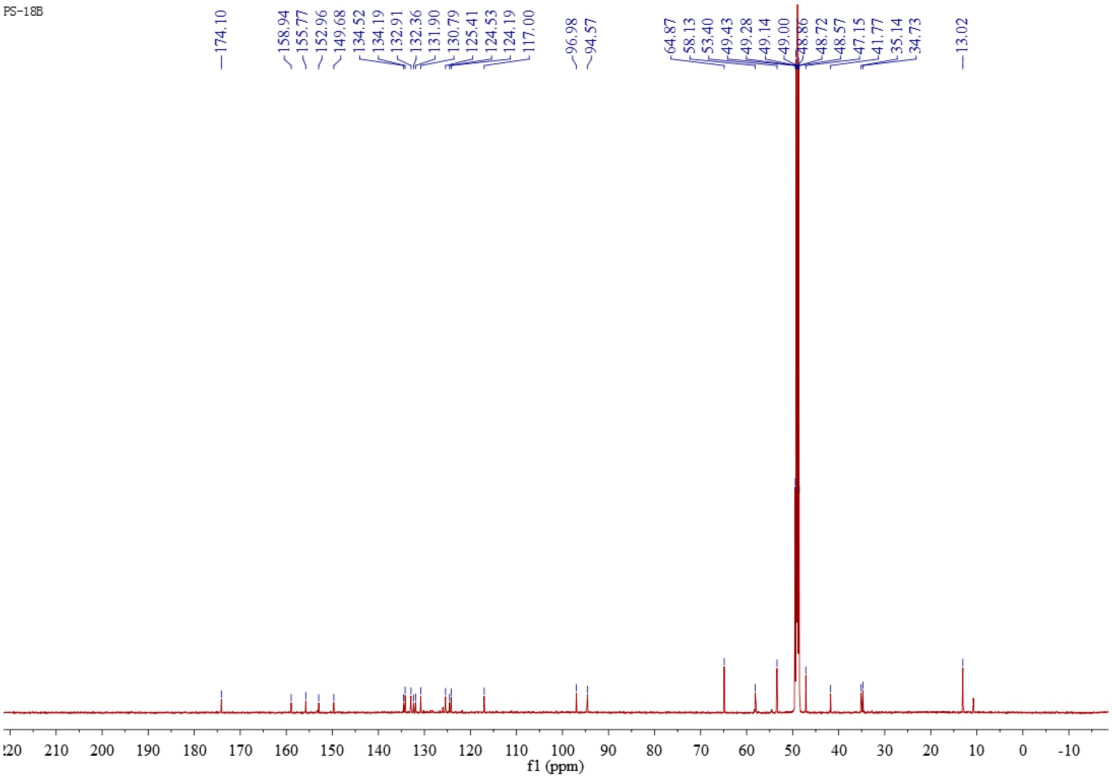

Figure 7. LR-MS spectrum of **PS-2**

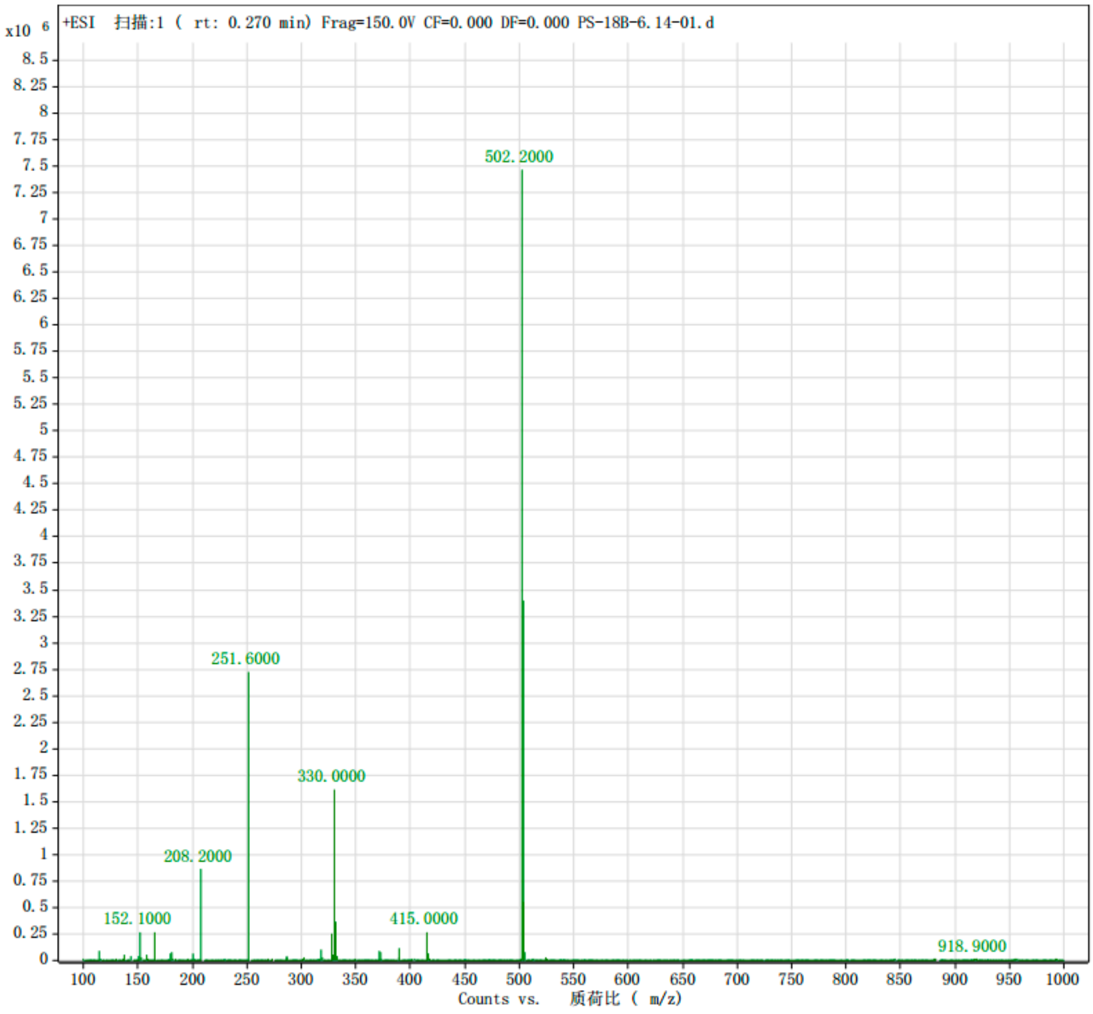

Figure 8. HR-MS spectrum of **PS-2**

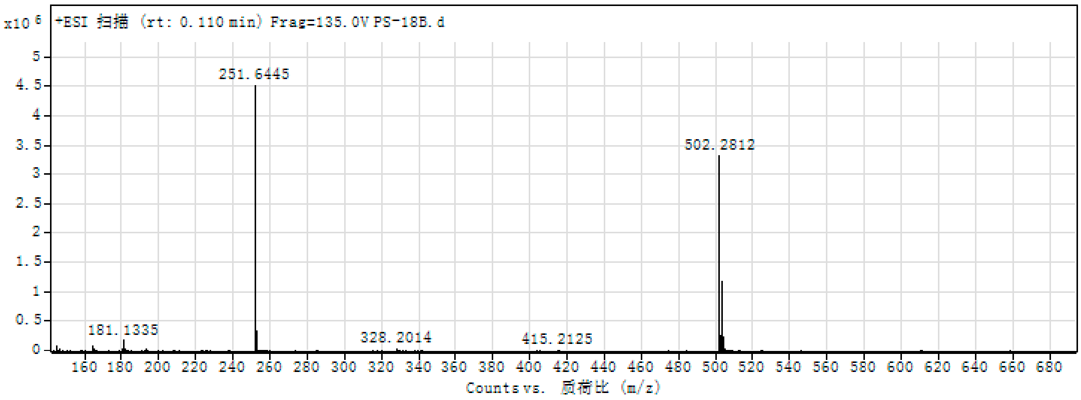

Figure 9. ^{1}H spectrum of **PS-3**

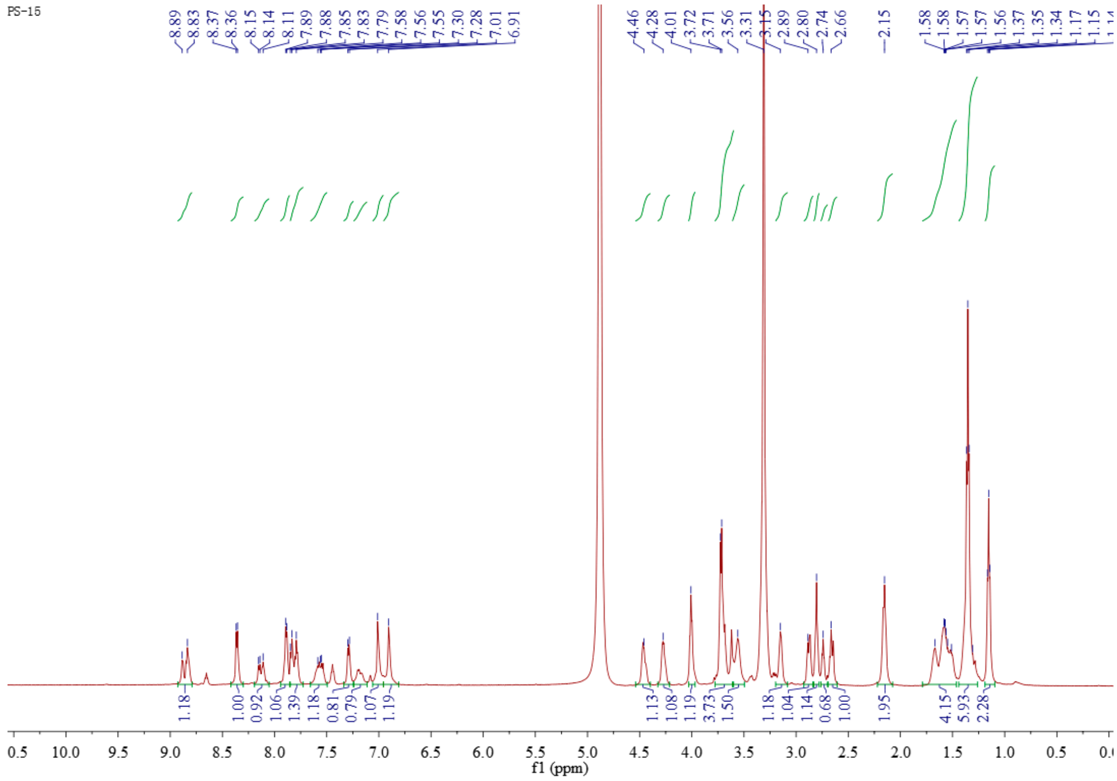

Figure 10. ^{13}C spectrum of **PS-3**

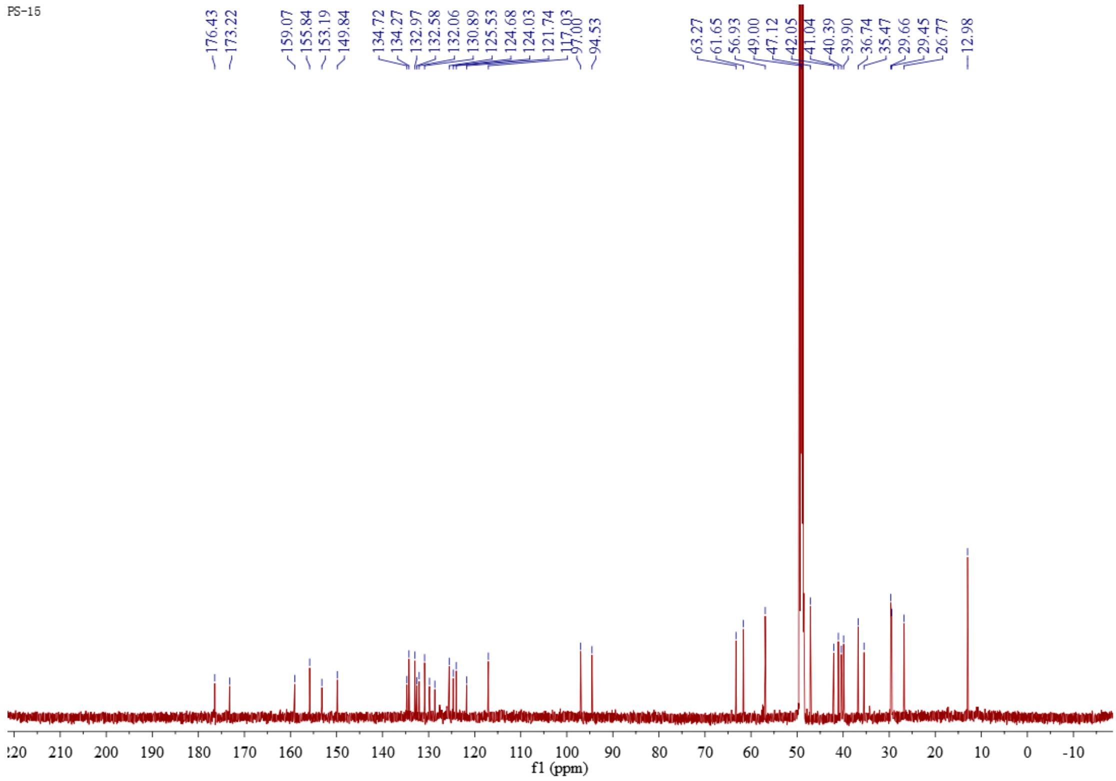

Figure 11. LR-MS spectrum of **PS-3**

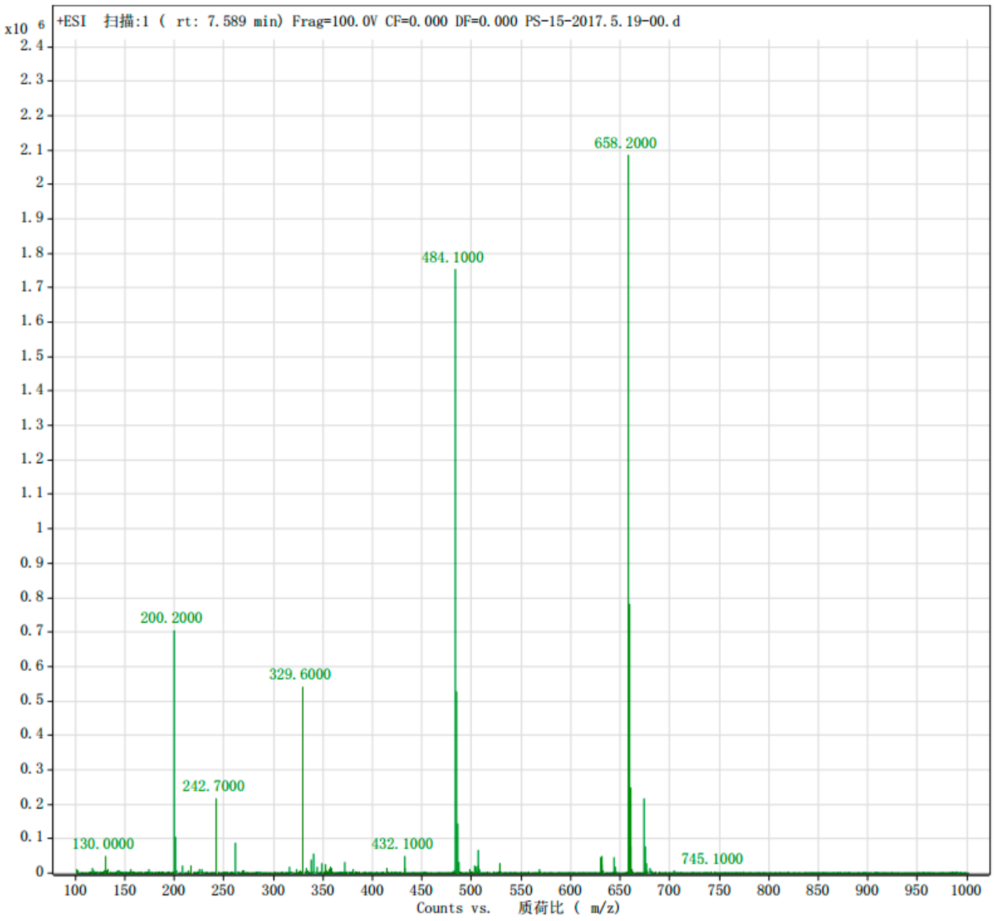

Figure 12. HR-MS spectrum of **PS-3**

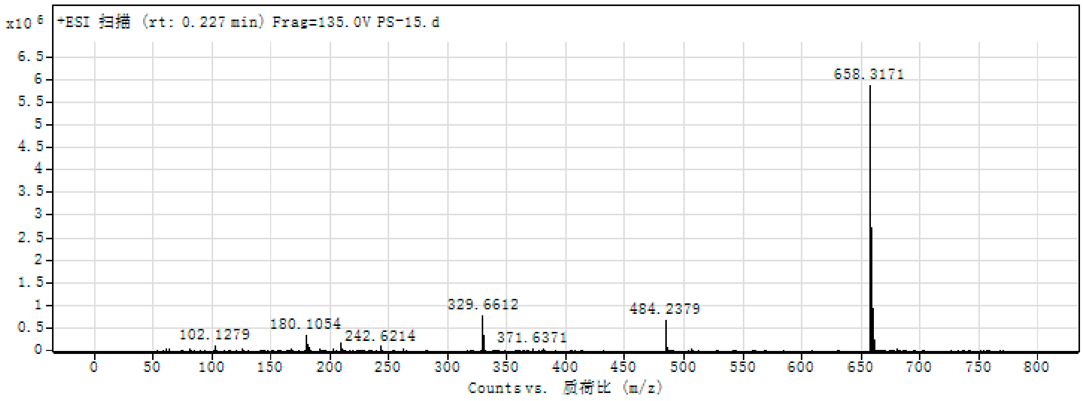

Figure 13. ^{1}H spectrum of **PS-4**

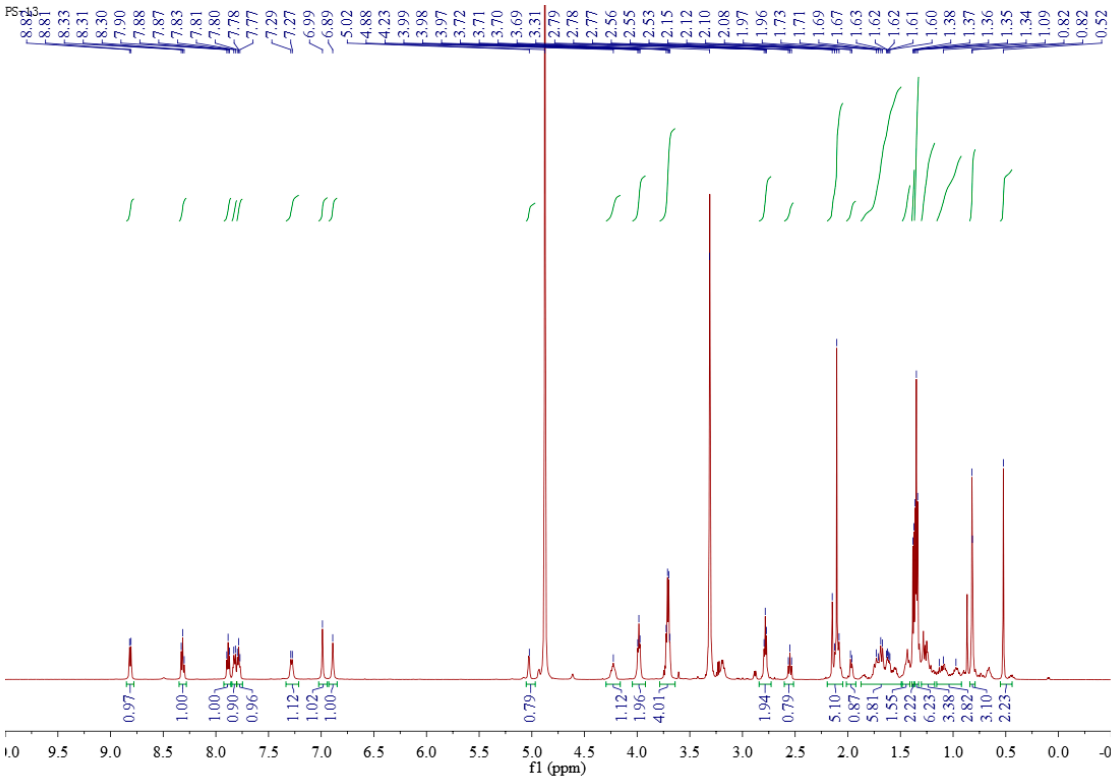

Figure 14. ^{13}C spectrum of **PS-4**

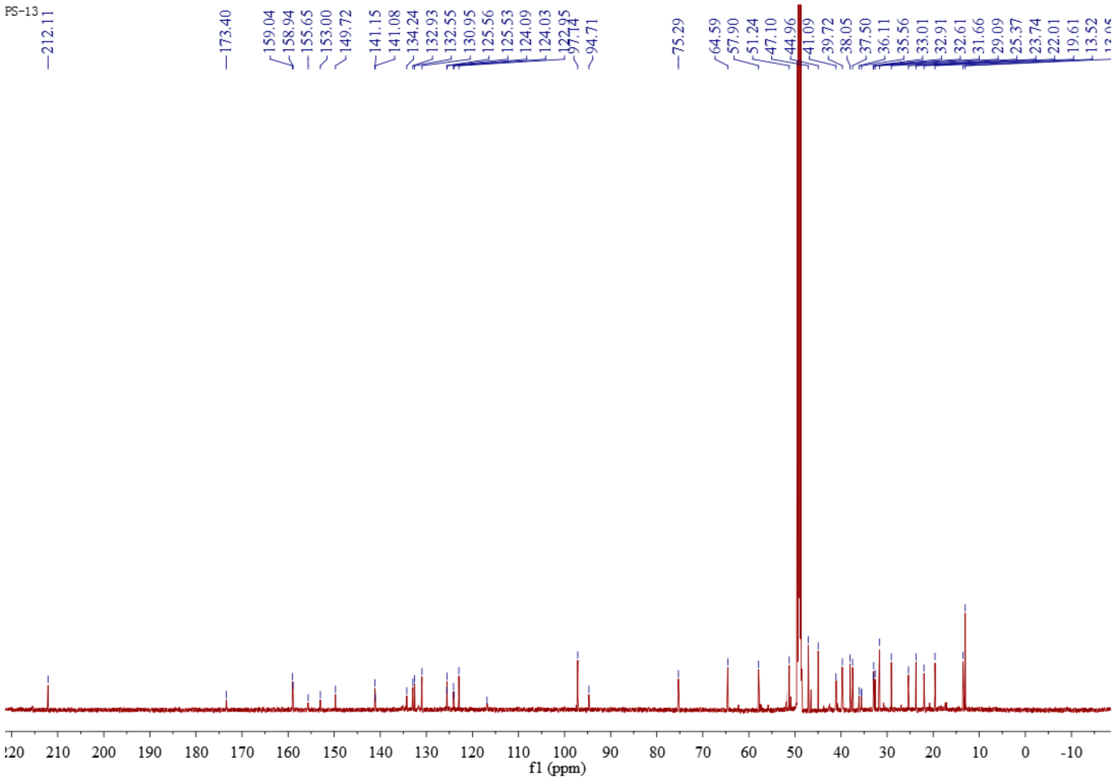

Figure 15. LR-MS spectrum of **PS-4**

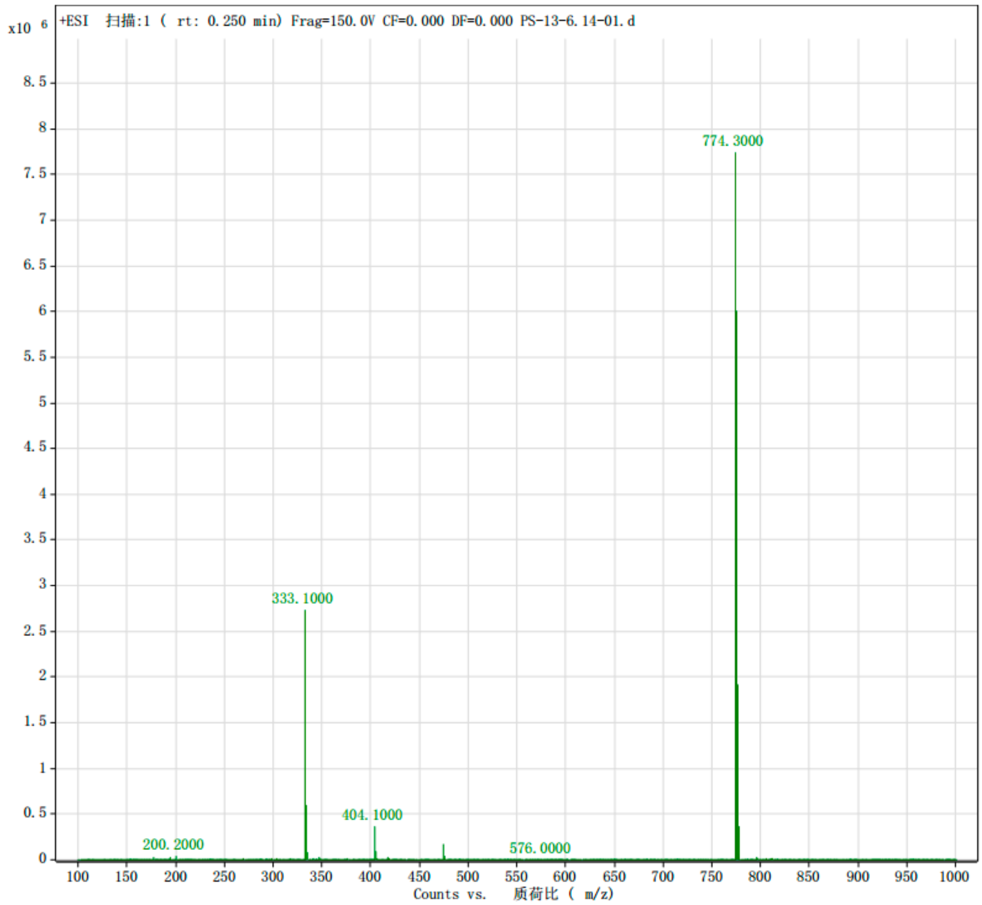

Figure 16. HR-MS spectrum of **PS-4**

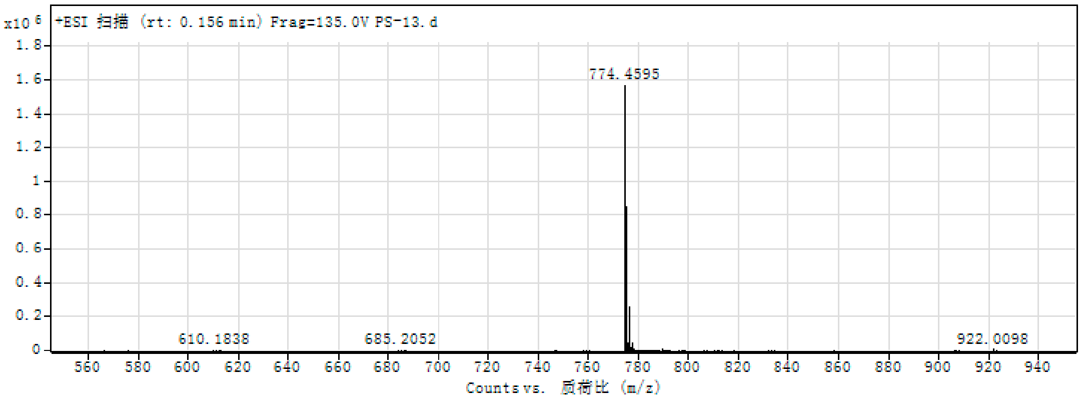

Figure 17. ^{1}H spectrum of **PS-5**

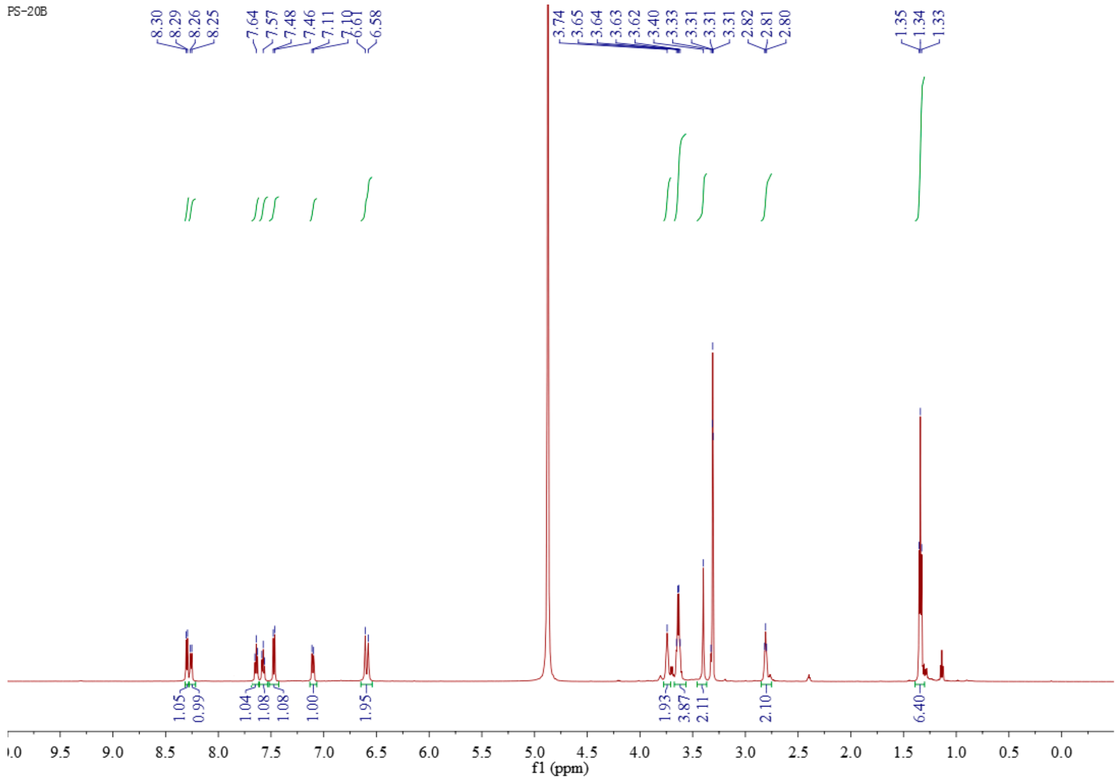

Figure 18. ^{13}C spectrum of **PS-5**

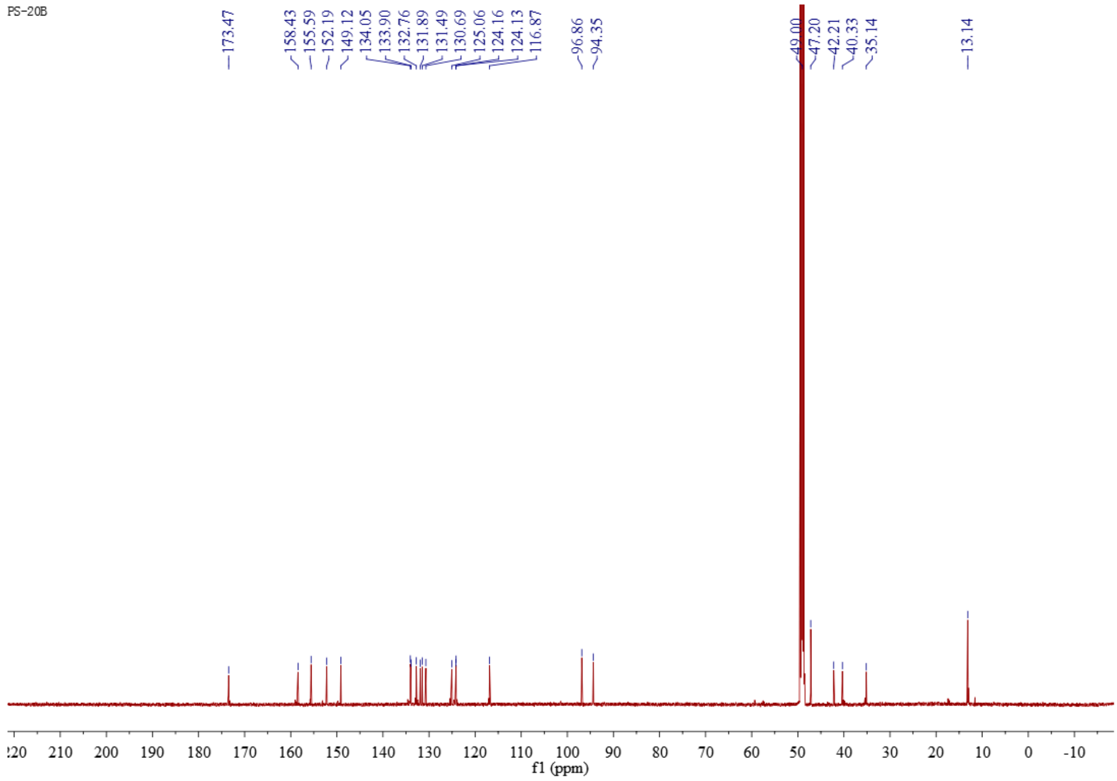

Figure 19. LR-MS spectrum of **PS-5**

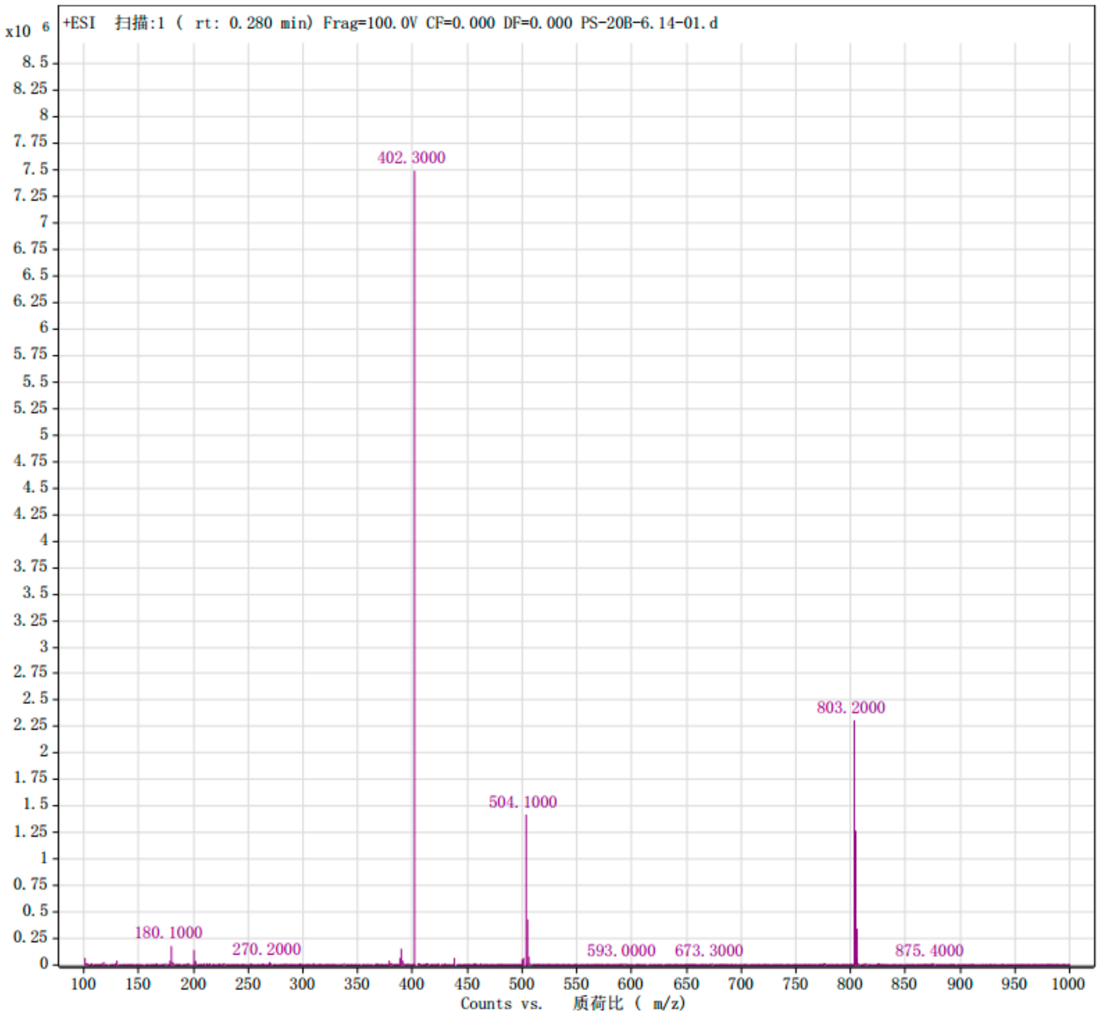

Figure 20. HR-MS spectrum of **PS-5**

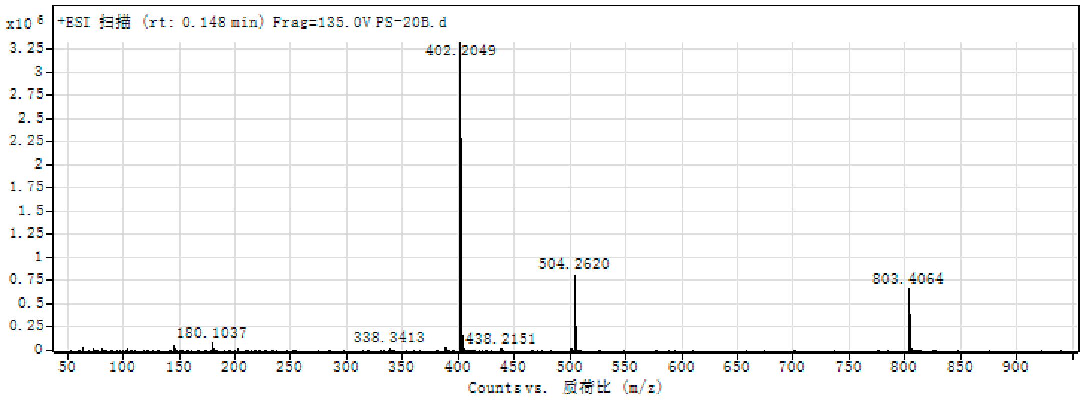

Supplement: Supplementary file 1 [file molecules-23-01436-s001.pdf]
